# Retraction: Exosomal miR-25-3p derived from hypoxia tumor mediates IL-6 secretion and stimulates cell viability and migration in breast cancer

**DOI:** 10.1039/d1ra90076e

**Published:** 2021-02-03

**Authors:** Laura Fisher

**Affiliations:** Royal Society of Chemistry Thomas Graham House, Science Park, Milton Road Cambridge CB4 0WF UK advances-rsc@rsc.org

## Abstract

Retraction of ‘Exosomal miR-25-3p derived from hypoxia tumor mediates IL-6 secretion and stimulates cell viability and migration in breast cancer’ by Zhengmin Li *et al.*, *RSC Adv.*, 2019, **9**, 1451–1459, DOI: 10.1039/C8RA06750C.

The Royal Society of Chemistry hereby wholly retracts this *RSC Advances* article due to concerns with the reliability of the data. The images in the article were screened by an image integrity expert. Many western blot panels have been over-contrasted to the point where the background has been almost erased. The remaining background, especially in Fig. 1, looks uncharacteristic and does not look genuine.

The authors were asked to provide the raw data for this article, but did not respond. Given the significance of the concerns about the validity of the data, and the lack of raw data, the findings presented in this paper are not reliable.

The authors were informed but have not responded to any correspondence regarding the retraction.

Signed: Laura Fisher, Executive Editor, *RSC Advances*

Date: 19^th^ January 2021

## Supplementary Material

